# Motor recovery patterns in arm muscles: coupled bilateral training and neuromuscular stimulation

**DOI:** 10.1186/1743-0003-11-57

**Published:** 2014-04-11

**Authors:** Nyeonju Kang, Jerelyne Idica, Bhullar Amitoj, James H Cauraugh

**Affiliations:** 1 Motor Behavior Laboratory, University of Florida, Gainesville, FL, USA; 2 Motor Behavior Laboratory, Applied Physiology and Kinesiology Department, University of Florida, Gainesville, FL 32611-8206, USA

**Keywords:** Stroke, Motor recovery, Coupled bilateral movements and neuromuscular stimulation, EMG Activation patterns

## Abstract

**Background:**

Neuromuscular stimulation coupled with bilateral movements facilitates functional motor recovery of the upper extremities post stroke. This study investigated electromyography activation patterns during training. The leading question asked: Do EMG activation patterns show rehabilitative effects of coupled bilateral movement training on wrist and fingers extension, elbow extension, and shoulder abduction?

**Methods:**

Twelve stroke volunteers completed nine hours of coupled bilateral movement training on three sets of joints in their arms. Neuromuscular stimulation on the impaired limb assisted wrist and fingers extension, elbow extension, and shoulder abduction. Mean activation level data were analyzed in a three-way completely within-subjects ANOVA (Training Day × Movement Type × Trial Block: 3 × 3 × 3).

**Results:**

The analysis revealed three important findings: (a) activation levels in Days 5 and 6 were significantly higher than Days 1 and 2, (b) muscle activation patterns increased across trial blocks, and (c) movements for the shoulder joint/girdle as well as wrist and fingers demonstrated higher activation than the elbow joint. Further analysis indicated that the muscle activation patterns for shoulder abduction were positively associated with force stabilization (ratio of good variability relative to bad variability) during bilateral force production.

**Conclusions:**

The findings indicate that capability to increase muscle activity during the three joint movements was improved after training. There appears to be higher muscle activation in the primary proximal and distal muscles necessary for motor control improvement.

## Background

Upper extremity hemiparesis is a common consequence of a stroke. Unfortunately, muscle weakness, partial paralysis, and involuntary movements disrupt many activities of daily living. Thus, individuals who experienced a stroke must complete an extensive amount of rehabilitation to overcome functional impairments in the arms. Indeed, rehabilitation protocols designed to re-learn basic arm movements are primary goals post stroke [1-3].

One effective rehabilitation protocol involves coupled bilateral movements and neuromuscular stimulation (i.e., coupled bilateral movement training) on the more impaired arm: the coupled bilateral movement training revealed more blocks moved, faster reaction time, greater force production, and higher peak limb velocity [2-7]. Subjects attempted to contract their impaired and weakened muscles while moving their other arm in the same motion. Surface electrodes attached to the weakened muscles and microcomputer monitored activation levels. Once the muscle activity reached a target intensity level, the microcomputer automatically provided neuromuscular stimulation and movement was executed. The coupled bilateral movement training helped the impaired muscles perform basic movements [2,3,5].

However, explicit details on the electromyography (EMG) activation patterns attained during the rehabilitation training are still unknown. Specifically, clarifying the role of voluntary muscle activation would help identify critical components of an effective protocol. The current upper extremity rehabilitation protocol focused on the motor recovery of three primary joint movements: (a) wrist and fingers extension, (b) elbow extension, and (c) shoulder joint abduction [2,3]. Comparing the EMG activation patterns among three joint movements may provide a fundamental rationale in determining specified optimal training intensity and frequency for each of the three joint movements. Thus, the primary objective of this study was to investigate the EMG activation levels during training to determine whether these patterns showed rehabilitative effects of coupled bilateral movement training on the three joint movements.

To determine effective muscle activation patterns during coupled bilateral movement training, measuring a response variable that represented both arms is necessary. Bilateral force control variability qualified as a response variable that estimates the effect of the bilateral training protocol because previous studies reported that stroke survivors showed reduced force variability after the training and the less variability was strongly associated with improved upper extremity functions (e.g., greater Fugl-Meyer Assessment score) [3,8]. Recent studies investigated the force variability based on the uncontrolled manifold hypothesis because all force variability does not always compromise task performance [8,9]. The uncontrolled manifold (UCM) hypothesis implies that the number of solutions to achieve a goal (i.e., a way to match bilateral force to the target force level) is infinite and uncontrolled in an abundant system. The UCM hypothesis postulates two sub-spaces in the two-dimensional Cartesian coordinate (x-axis: right hand force; y-axis: left hand force): (a) UCM line (i.e., perfect performance) and (b) ORT line (i.e., orthogonal to the UCM line). Thus, variability of the fundamental components, bilateral forces, projected onto the UCM line is defined as good variability contributing to bilateral force control whereas variability of components projected onto the ORT line is defined as bad variability impeding force control in bilateral movements. The proportion of the good variability to the bad variability indicates motor synergies, and this measure is associated with stabilization of performance (force control) [9,10]. These findings lead to an additional question focused on the tenets of the UCM hypothesis: Are muscle activation patterns during the three joint movements associated with the force stabilization index after a rehabilitation protocol?

## Methods

### Participants

Twelve chronic stroke patients (age = 65.1 ± 17.6 years; 6 males and 6 females) volunteered to participate. The participants were recruited from North Central Florida; stroke groups and rehabilitation facilities by information on announcements and bulletin boards as well as word-of-mouth. Four inclusion criteria follow: (1) unilateral stroke experienced more than 6 months before entering the study; (2) ability to voluntarily activate a NeuroMove™ microprocessor unit for neuromuscular stimulation; (3) voluntary wrist and fingers extension movement from a 80° flexion position to 10° extension position, elbow extension movement (145° − 0°), and shoulder abduction movement (0° − 90°); and (4) unimpaired cognitive capacity (Mini-Mental State Examination score > 23) [11]. Excluded participants had additional neurological or musculoskeletal deficit, visual and auditory disorder, or orthopedic injury pain in their upper extremities. Clinical information for participants is shown in Table 1. According to the Stroke Impact Scale (version 3.0), participants were relatively mildly impaired [12]. All participants read and signed an informed consent form approved by the Institutional Review Board at the University of Florida before testing and rehabilitation training began.

**Table 1:** Characteristics of the stroke participants

**Subject no.**	**Age (Year)**	**Sex**	**Stroke type**	**Affected Hemisphere**	**Time since stroke (Month)**	**Stroke impact scale (Upper extremity function)**
1	72.7	F	I	R	84	22
2	66.3	M	I	L	41	20
3	78.8	F	I	R	60	16
4	80.4	M	I	R	6	24
5	20.0	M	H	L	13	16
6	50.8	F	H	R	10	5
7	63.0	M	I	L	7	21
8	79.8	M	I	L	8	17
9	76.7	F	I	R	19	21
10	51.0	F	H	R	13	13
11	76.8	F	I	R	20	23
12	64.8	M	I	L	18	9
Total	65.1 ± 17.6	6 F/6M	9I/3H	5L/7R	24.9 ± 24.4	17.3 ± 5.8

Mean ± Standard deviation; M: male; F: female; I: ischemic; H: hemorrhagic; L: left; R: right; 5 items for upper extremity function domain in the Stroke Impact Scale (scores for each item: 1, 2, 3, 4, and 5; higher score indicates improved motor function); score range for upper extremity function from 0 to 25 (higher score indicates improved motor function).

### Rehabilitation protocol: neuromuscular stimulation coupled with bilateral movement

Consistent with previous coupled bilateral movement training studies, the rehabilitation protocol involved movements on both arms in combination with EMG-triggered neuromuscular stimulation on three sets of impaired muscles [3,5,8,13]. Optimal training intensity levels involving duration and frequency are crucial for effective rehabilitation programs. In previous rehabilitation studies, the duration of neuromuscular stimulation varies between 30 to 60 minutes one to three times per day for two weeks up to three months [6,14,15]. For the current study, the microcomputer monitored target threshold ensuring that participants were continually challenged to increase their muscle activation levels to new target areas. Further, the rehabilitation sessions lasted approximately 90 minutes per day, one day per week for six consecutive weeks.

The joint movements were wrist and fingers extension, elbow extension, and shoulder abduction. For the elbow joint and shoulder joint movements, participants had to overcome slight resistance provided by therapists because of two reasons. First, the shoulder joint has many large muscles surrounding the joint. Slight resistance ensured that activating proximal movements involved challenging efforts. Second, elbow extension was performed with gravity (toward the table). Again, slight resistance ensured EMG activation of the proximal muscles. Further, the resistance provided by therapist was over the full range-of-motion for both joint movements. Participants were able to recruit more motor units than without any resistance. This was consistent with previous studies [5,16]. Further, three sets of the three upper extremity movements were presented randomly (i.e., 3 sets × 3 movements × 10 trials per set = 90 trials each day) and participants did not perform two consecutive sets for same joint movement.

**Figure 1:**
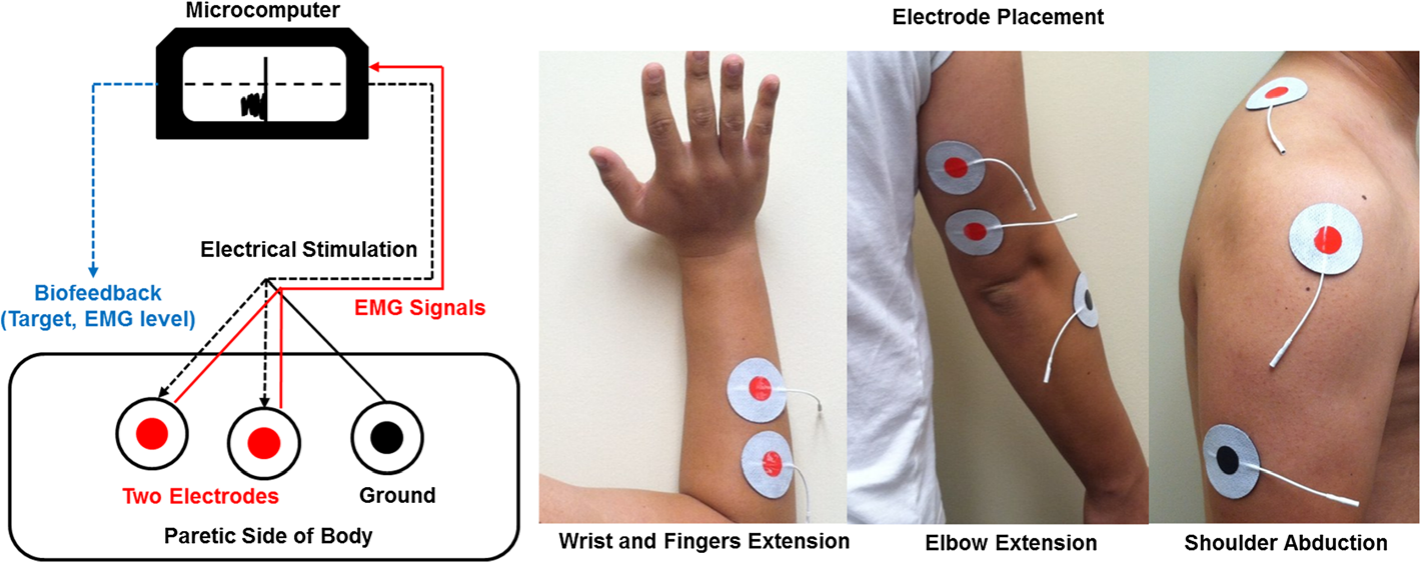
Experimental setup for providing electrical stimulation and recording EMG activation.

EMG-triggered neuromuscular stimulation assisted voluntary movement initiation in the upper extremity [3,5,17]. During the coupled bilateral movement training, therapists consistently attached electrodes across sessions based on prominent anatomical bases. After cleaning the skin, two EMG signal/stimulation electrodes (5.08 cm diameter each) were attached to the agonist muscles (primary movers), and a ground electrode was attached to an indifferent muscle (not an agonist or antagonist) in another region of the arm (Figure 1). The electrodes and microcomputer detected EMG activity in the primary muscles, and once the signals reached a threshold level, the unit and electrodes served as stimulators providing electrical stimulation to the motor end-points (i.e., muscle belly; middle of the muscle) for a maximum range of motion [6]. Therapists maintained these placement procedures throughout training. The agonist muscles for three joint movements involved: (a) extensor communis digitorum and extensor carpi ulnaris muscles for wrist and fingers extension, (b) triceps brachii muscles for elbow extension, and (c) middle deltoid and trapezius (supporting upward rotation of the scapula) muscles for shoulder abduction. Consistent with previous studies [3,5,13], standardized NeuroMove™ settings included: (a) an initial threshold level of 50 μV, (b) stimulation frequency at 50 Hz, (c) 5 s stimulation period, (d) pulse width of 200 μs, (e) 1 s ramp-on and 1 s ramp-off, and (f) 15 s of rest between trials. The target threshold levels consist of 56 stages. The microprocessor unit automatically increased the level of threshold to the next stage when an individual’s EMG activity exceeded the target level on two consecutive trials. On the other hand, if participants did not reach the target level, then the microprocessor unit decreased the threshold level to the previous target level. The threshold level from the last trial of the previous day was used to begin each training day. For each trial, the microcomputer automatically detected peak rectified EMG activation values (μV) in the muscle signals. These values reflected patterns of small EMG changes (i.e., effort) instead of averaging the input. Given that many stroke survivors display hemiparesis and are dysfunctional, clean integrated EMG signals are challenging to obtain [18]. EMG activation levels (μV) were recorded manually for all successive movements.

### EMG activation data analysis

**Figure 2:**
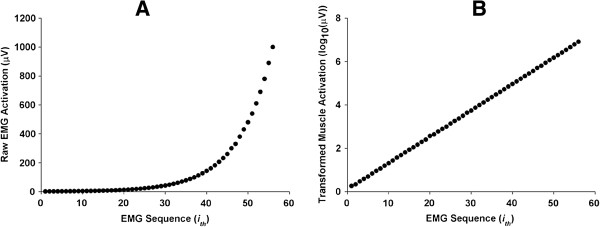
**Distribution of raw muscle activation and transformed muscle activation.** Each EMG sequence is a successively higher level than the previous level. **A**. Raw muscle activation levels (*n* = 56) provided by microprocessor unit (exponential and heavily skewed). **B**. Transformed muscle activation levels (*n* = 56) with log_10_N (linear).

Given that the voluntary EMG activation patterns were skewed, the data were transformed by log_10_N [18]. Figure 2 shows an individual’s original data and transformed values. The transformed (log_10_) data were used to calculate means and standard deviations for training days, movement types, and trial blocks. Based on previous studies [18,19], data for each training day and trial were grouped to identify general trends. The six training days were grouped as three sets of two days each (i.e., Day 1 and 2; 3 and 4; 5 and 6). Creating three separate blocks of means for training days allowed a direct comparison of early, middle, and later training. The three movement types were wrist and fingers extension, elbow extension, and shoulder abduction. Further, the trial block means represented the three sets of 10 trials completed during training (i.e., block 1: 1–10; block 2: 11–20; block 3: 21–30). Three separate blocks of means for trials allowed a robust examination of changes across the trials [18]. The transformed means of the muscle activation patterns were analyzed in a three-way repeated measures ANOVA (Training Day × Movement Type × Trial Block: 3 × 3 × 3). Tukey-Kramer’s test was used for post hoc analysis when any significant effect was identified in the three-way repeated measures ANOVA. All statistical tests were conducted with alpha level set at 0.05.

### Bilateral force control: wrist and fingers extension

Wrist and fingers extension movements for isometric force control were tested because moving the wrist and fingers for isometric force control task was strongly associated with conventional clinical measurements [20]. Moreover, stroke survivors showed more deficits in the distal movements of wrist and fingers involved in force control than age-matched controls [20,21]. Being able to overcome a flexor synergy in the wrist and fingers is critical to motor improvements post stroke. The bilateral force control task was administered one week after the last training to avoid any fatigue effects at the end of the last day of training. Given that the total force is the sum of two forces from each hand (i.e., Force_TOTAL_ = Force_LEFT_ + Force_RIGHT_) while executing isometric wrist and fingers extension on both hands simultaneously, the force variability structure was calculated using the uncontrolled manifold hypothesis [9,22]. One of the tenets of this hypothesis involves two components of force variability: (a) good variability, a positive element of variability that assists force control, and (b) bad variability, a negative element of variability that impedes force control. The ratio of these two variability elements served as the converging operations approach in determining motor capabilities post stroke.

The participants were seated in front of a 43.2 cm monitor located 78 cm away at eye level. Standardizing wrist and fingers joint position during the force control, participants placed their left and right forearms on the table with 15 − 20° of shoulder flexion and 20 − 40° of elbow flexion. Further, the hands and fingers of both arms were placed under a padded platform that contained force transducers. Platform height was adjusted to accommodate individual hand thickness for each participant and this ensured initial extension of the wrist and fingers pressed against the platform and registered on the load cells. This position was consistent across all participants.

**Figure 3:**
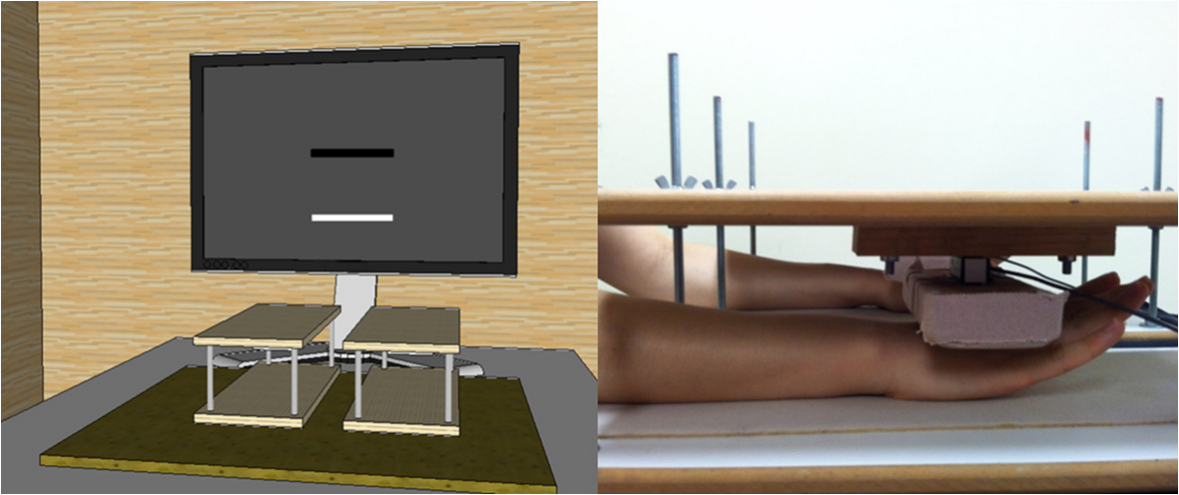
Bilateral force control task and position for wrist and fingers extension.

Before testing began, two maximum voluntary contraction (MVC) trials were performed during bilateral movements. These MVC values indicate the maximum level of combined bilateral force. Consistent with a recommendation that force control of 25% MVC provided an objective and meaningful assessment [20,23], participants performed three trials at the submaximal force level. Visual information was used to match their total force to the 25% of MVC target force. To begin a trial, participants viewed a stationary black bar (e.g., target force; 256 × 20 pixels) and a movable white bar (e.g., bilateral force production; 256 × 20 pixels; visual gain = 50 pixel/N). The 20 s task required participants to extend their wrists and fingers upward against the padded platform as they attempted move the white bar on top of the black bar (Figure 3).

Two load cells (MLP-75, Transducer Techniques, 4.16 × 1.27 × 1.90 cm, range = 75 lbs, 0.1% sensitivity) attached to the platforms collected the force data. The force output from each load cell (hand) was amplified by a 15LT Grass Technologies Physio-data Amplifier System (Astro-Med Inc.) with an excitation voltage of 10 V and a gain of 200. Sampling rate was 100 Hz using a 16-bit analog-to-digital converter (A/D; NI cDAQ-9172 + NI 9215) that detects force unit minimally 0.0016 N. A bidirectional fourth-order Butterworth filter with cut off frequency set at 20 Hz filtered the force data. Data acquisition was conducted on a custom LabVIEW Program (National Instruments, Austin, USA). The force data were saved and submitted to a custom Matlab program (Math Works™ Inc., Natick, Massachusetts, USA) for offline analysis.

### Force control analyses

**Figure 4:**
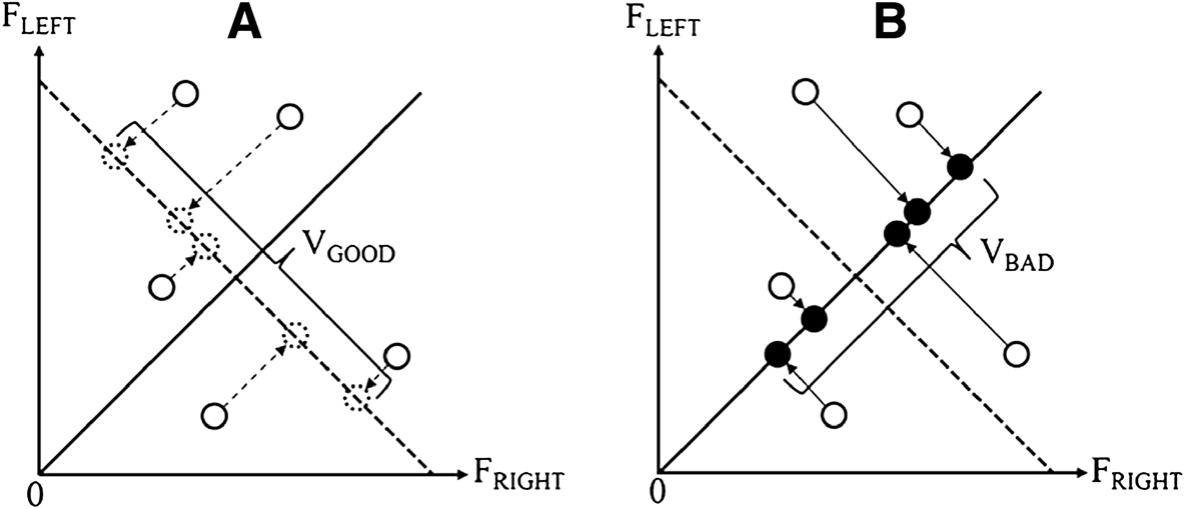
**Good and bad variability for total force simultaneously produced by the left and right hands. A**. Calculation of V_GOOD_ using projection of total force onto dashed line (i.e., UCM line). **B**. Calculation of V_BAD_ using projection of total force onto orthogonal line (i.e., ORT line) to the dashed line.

To remove initial force adjustment and early termination effects, 14 s of middle force signals were used for data analyses (e.g., first 5 s and final 1 s of force data eliminated) [8,20]. For force amplitude, force output means during bilateral force control at 25% of MVC were calculated for each trial. Further, to determine whether the force control task was performed bilaterally, force asymmetry (i.e., ratio of paretic hand force to bilateral force) was computed for each trial. The two components of force variability from the bilateral force task, consistent with the uncontrolled manifold hypothesis, were calculated within a single trial using the two formulas below (1 and 2) [8,22,24]. Figure 4 displays the amount of good variability (i.e., V_GOOD_; variability of elements projected onto the dashed line in Figure 4A; Formula 1) and bad variability (i.e., V_BAD_; variability of elements projected onto an orthogonal line to the dashed line in Figure 4B; Formula 2). In this study, bilateral forces (i.e., total force) represented fundamental elements and these values were projected onto the UCM line (i.e., uncontrolled manifold line: a dashed line) and ORT line (i.e., orthogonal line to the UCM line), respectively. Two variances of elements on both UCM line and ORT line were computed (e.g., good variability from the UCM line and bad variability from the ORT line).

(1) $$V_{\mathrm{GOOD}}=\frac{\sum {\left(F_{G}-\bar{F_{G}}\right)}^{2}}{N-1}$$

(2) $$V_{\mathrm{BAD}}=\frac{{\sum \left(F_{B}-\bar{F_{B}}\right)}^{2}}{N-1}$$

*F*_*G*_ represents a combined bilateral force at each time sample projected onto the UCM line and $\bar{F_{G}}$ is mean of the *F*_*G*_. Similarly, *F*_*B*_ represents an combined bilateral force at each time sample projected onto the ORT line and $\bar{F_{B}}$ is mean of the *F*_*B*_. N is total number of elements.

The ratio of the two variability components (R_V_ = V_GOOD_ / V_BAD_) represents an index of the stabilization of force production [9,22]. If the R_V_ is greater than 1, the force production is bilaterally stabilized and coordinated in a synergetic way. In contrast, if R_V_ is less than 1, the error of force output may increase because of the destabilization of force production and weak synergy. The optimal regression models used to predict the force amplitude and stabilization of force production from the level of muscle activation in the three movement types (Significant Level Entry; SLE = 0.08) were a stepwise multiple regression model [25-28]. A measure of goodness-of-fit of the model was the coefficient of determination.

## Results

### EMG activation patterns during training

**Figure 5:**
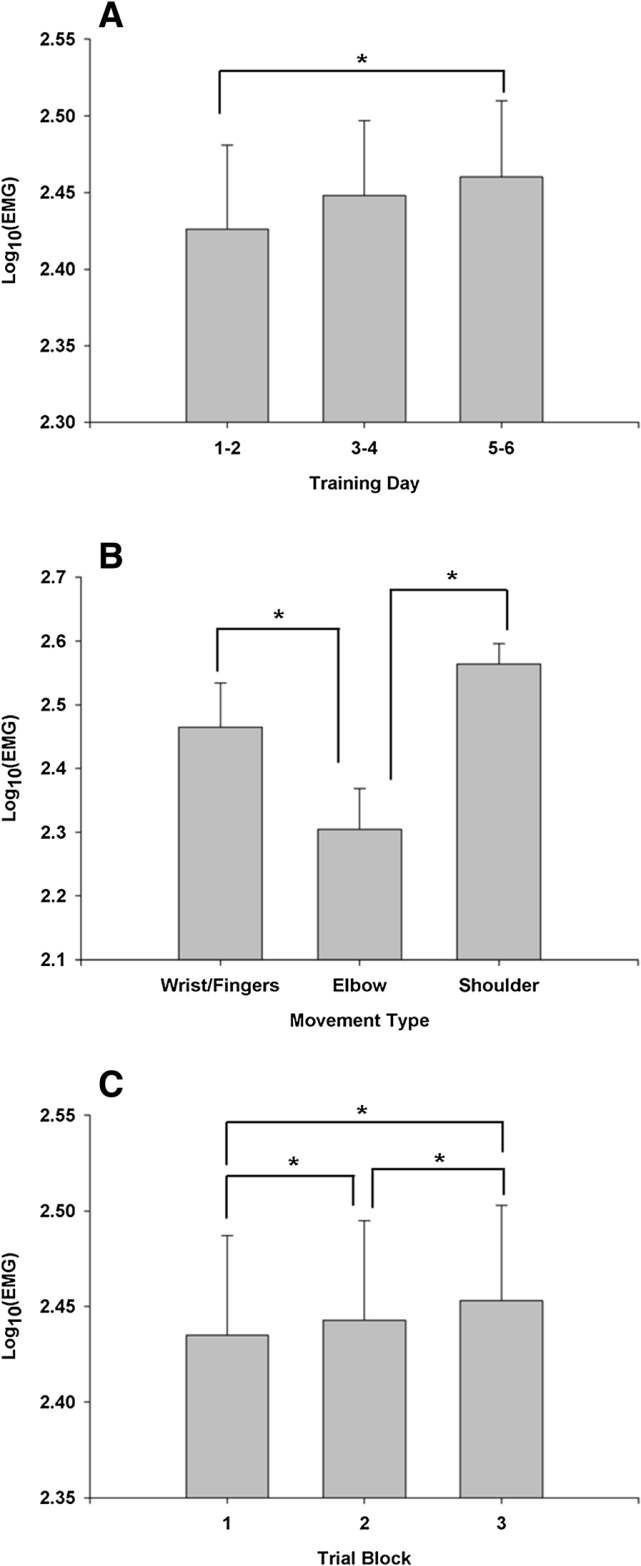
**Quantitative measure of EMG activation levels. A**. Training day main effect for transformed EMG activation levels (*M* ± *SE*). **B**. Movement type main effect for transformed EMG activation levels (*M* ± *SE*). **C**. Trial block main effect for transformed EMG activation levels (*M* ± *SE*). *Asterisk* (*) indicates significant difference (*p* < 0.05).

The completely within-subjects Training Day × Movement Type × Trial Block (3 × 3 × 3) ANOVA revealed three significant main effects. The training day main effect [*F*(2, 22) = 9.44; *p* = 0.01; partial η^2^ = 0.46] indicated that the EMG activation levels significantly increased across the days (see Figure 5A). Tukey-Kramer’s follow-up procedure revealed that the activation level on the third set of days (5 and 6) than on the first set of days (1 and 2). However, the activation level on the third set of days was not significantly greater than on the second set of days.

The significant movement type main effect [*F*(2, 22) = 17.29; *p* < 0.01; partial η^2^ = 0.61] is displayed in Figure 5B. Follow-up analysis indicated more EMG activity for wrist and fingers extension and shoulder abduction movements than elbow extension. Under the same intensity of training for the three joint movements, the EMG activation level in elbow extension movement was less than the other two joint movements across training days.

The third reliable main effect was trial block [*F*(2, 22) = 31.663; *p* < 0.01; partial reliable main effect was ^2^ = 0.74]. Post hoc analysis revealed higher values of muscle activation for the second trial block versus the first trial block. Further the trend continued in the third block, a higher activity level than the first and second trial blocks. As seen in Figure 5C, the trial block findings display continual increases in neuromuscular activation levels across the coupled bilateral movement training.

### Force variability in bilateral movements

**Figure 6:**
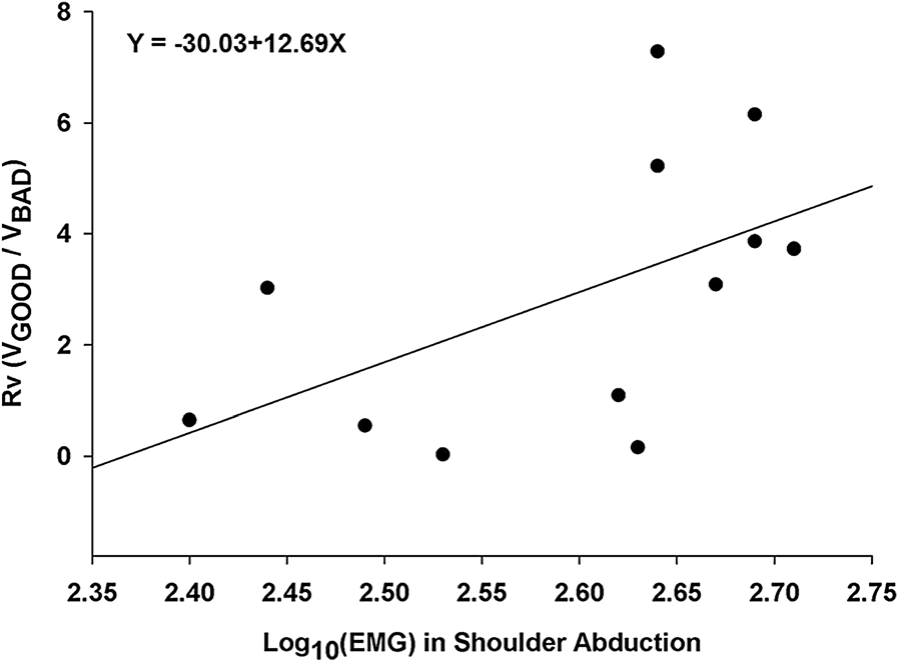
Prediction of the index of force stabilization from the muscle activation patterns during shoulder abduction.

The force asymmetry (paretic hand force/bilateral force × 100) mean equalled 42.3% (*SE* = 4.5%). This indicated that total force outputs were relatively symmetrically produced from both hands. Additional analyses compared the muscle activation patterns for the three types of movement and the force amplitude and index of stabilization during bilateral force production. To determine whether a meaningful relationship between the muscle activation patterns and force amplitude (i.e., mean force outputs at 25% of MVC) and index of the stabilization of force control (i.e., R_V_ = V_GOOD_/V_BAD_) existed multiple linear regression analyses were performed using stepwise regression (SLE = 0.08). Specifically, the explanatory variables involved the EMG activation levels for the Trial Block 3 of the Day 5 and 6 for each movement type. The stepwise regression analyses revealed a strong trend in the level of muscle activation generated in the shoulder joint and girdle with the index of the stabilization of force production (Y = −30.03 + 12.69X, *R*^2^ = 0.29, *r* = 0.54, *p* = 0.07; see Figure 6). However, the analyses did not show a significant relationship between the muscle activation patterns and force amplitude. The optimal regression model revealed that as the levels of EMG activation in the deltoid and trapezius muscles increased, bilateral force production became more stable and coordinated.

## Discussion

The primary purpose was to investigate motor recovery patterns of three specific upper extremity joint movements during coupled bilateral movement training. The leading question asked: Do EMG activation patterns show rehabilitative effects of coupled bilateral movement training on three primary joint movements of impaired arms? The identified significant main effects for training days and trial blocks provided strong support for answering the above question. Later in training, participants were able to generate higher levels of muscle activation as intentional input in initiating arm movements during the coupled bilateral movement training. Further, increased intensity of the EMG activation patterns were found for both proximal and distal joint movements. Collapsed across the trial blocks and training days shoulder abduction and wrist and fingers extension exhibited increased activation levels.

Possible physiological mechanisms underlying the higher EMG activation patterns for joint movements in the shoulder and wrist and fingers involve more motor units (i.e., a motor unit = a motor neuron + all muscle fibers) activated and/or a higher firing rate of muscle fibers. As muscle contractions increase, more motor neurons and muscle fibers were activated [29-31]. Further increases in muscle activity are a function of firing rate [32,33]. Indeed, motor unit recruitment and firing rate of muscle fibers are associated with higher levels of force production [34]. The current findings suggest that the wrist and fingers and the shoulder may recruit more motor units and show higher firing rates of muscle fibers than the elbow during training. Perhaps these muscle activation patterns produced differential force for each of the three joint movement types.

Further, the significant training days and trial blocks were similar to earlier findings [2,5,17]. From a neuromuscular intensity perspective, these two main effects showed that the coupled bilateral movement training protocol continually challenged the stroke patients to contract more muscle fibers. Moreover, as rehabilitation progressed, the higher intensity levels indicate that participants learned to increase their EMG activation levels [18]. Thus, motor re-learning in the upper extremity appeared after training in this group of individuals who experienced a stroke.

In addition, controlling distal motor activity after a stroke depends on the simultaneous activation of proximal muscles in stabilization. Hoffmann et al. reported that the maximum productions of isometric finger extension and flexion torque were strongly influenced by co-activation of proximal and distal muscles [35]. Given that an ability to produce greater maximum force production was associated with improvements in force control at 25% of MVC [36], co-activation of the proximal and distal muscles may improve force control capabilities at the submaximal target force level. Thus, the present findings of higher activation patterns during wrist and fingers extension and shoulder abduction across training may contribute to the distal motor function of this stroke group.

Further, support on the importance of proximal muscle activation for the modulation of distal joint movements was found in the optimal linear regression model. For the force control task performed by wrist and fingers extension (e.g., distal joint movement control), the levels of muscle activation for shoulder abduction (e.g., proximal muscles) was positively associated with the index of force stabilization. Specifically, the proportion of good variability (i.e., elements that contribute) during force control increased with higher EMG levels for shoulder abduction. These results were consistent with previous findings concerning the function of shoulder abduction in the paretic limb; shoulder abduction was strongly correlated with hand function in stroke patients [37-39]. The implications of these results are that coupled activity of the proximal and distal muscles (i.e., intra-limb coupling) is crucial for successful hand movements in stroke survivors. Indeed, coupled activity for both targeted muscles and untargeted muscles during training sessions may contribute to more improved force control capabilities in comparison to an isolated joint movement for targeted muscles.

## Conclusions

This study showed that coupled bilateral movement training increased EMG activation patterns in three primary joint movements during training. The EMG activation levels involved in wrist and fingers extension and shoulder abduction were greater than elbow extension. Further, consistent with stroke motor impairments [39], planned hand functions (e.g., wrist and fingers force control) are most likely affected by proximal as well as distal muscle activation. Therefore, rehabilitation therapists should consider a program like coupled bilateral movement training as a way to improve motor capabilities for proximal and distal muscles. Both sets of muscles may contribute to positive rehabilitative effects on hand functions in stroke patients. Indeed, given that the current upper extremity functions were relatively mildly impaired, we limit our generalizability to mildly impaired individuals. Thus, in future studies, we intend to investigate EMG activation patterns for the three primary joint movements in individuals with moderate to severe upper extremity functions [4,5].

## Abbreviations

EMG: Electromyography; MVC: Maximum voluntary contraction; SLE: Significant level entry; UCM: Uncontrolled manifold.

## Competing interest

The authors declare that they have no competing interests.

## Authors’ contributions

NK contributed to statistical analyses, data interpretation, manuscript writing, and revisions. JI and BA were involved in data collection and manuscript revision. JHC conceived and designed the study, conducted statistical analyses, wrote and approved extensive revisions for the final manuscript. Each author has read and approved the final manuscript.
